# Extensive Postpartum Upper and Lower Extremity Deep Vein Thrombosis in a Patient With a History of Preeclampsia

**DOI:** 10.7759/cureus.41109

**Published:** 2023-06-28

**Authors:** Nolberto Jaramillo, Anisa Raidah, Katherine F Pradas, Steven Lev, Anantha Ramanathan

**Affiliations:** 1 Surgery, New York Institute of Technology College of Osteopathic Medicine, Old Westbury, USA; 2 Surgery, Stony Brook University, Stony Brook, USA; 3 Neuroradiology, Nassau University Medical Center, East Meadow, USA; 4 Surgery, Nassau University Medical Center, East Meadow, USA

**Keywords:** preeclampsia, acute pulmonary embolism, acute deep vein thrombosis, post-partum period, upper extremity thrombosis, pre-eclampsia

## Abstract

We describe the case of a 19-year-old woman with no significant medical history who developed progressive right-sided neck pain and palpitations one month following a pregnancy complicated by preeclampsia. Family history was significant for unprovoked deep vein thrombosis (DVT) and pulmonary embolism (PE) in her father at age 44. Systemic examination revealed mild swelling of the right upper extremity with pain on palpation. Computed tomography (CT) of the thorax with contrast demonstrated extensive occlusion of right upper extremity veins and collateralization of chest wall veins. Pulmonary emboli were present bilaterally in the segmental and subsegmental branches of the lower lobe pulmonary arteries. CT of the abdomen with contrast revealed thrombi in the left common and external iliac veins. Thrombophilia screening was normal. The patient was treated with enoxaparin and ampicillin/sulbactam. Her clinical condition improved, and she was discharged with an outpatient clinic follow-up appointment.

## Introduction

Pregnancy is a known risk factor for deep vein thrombosis (DVT), the highest risk being in the early postpartum period [1]. Preeclampsia and cesarean section further increase the risk of DVT [2]. Lower extremity DVT (LEDVT) is the most common type of venous thromboembolism (VTE) in the postpartum period, although pulmonary embolism (PE) and upper extremity DVT (UEDVT) may also occur. UEDVTs are rare (accounting for 4% of DVTs) and are associated with central venous catheters, various prothrombotic states, and strenuous activity with the upper extremity [3]. Very few cases of UEDVTs have been reported in the postpartum period, especially in association with extensive LEDVT and PE [4].

## Case presentation

**Figure 1:**
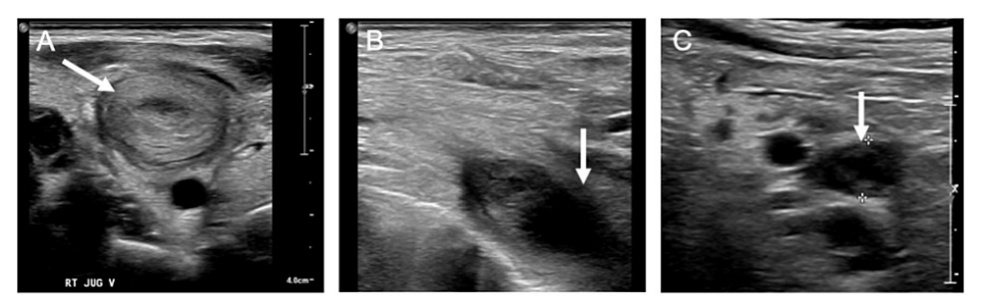
Duplex ultrasound of the upper extremity veins revealing diffuse deep vein thrombosis. Intraluminal echogenicity indicating thrombus (white arrow) in the right internal jugular vein (A), subclavian (B), and axillary (C).

A 19-year-old Hispanic female, gravida 1 para 1, presented to the emergency department with a three-day history of right-sided neck pain and palpitations. The patient had been evaluated earlier that day in the obstetrics and gynecology clinic for follow-up of a cesarean section performed one month prior for a pregnancy complicated by severe preeclampsia and anemia. There was no significant past medical history aside from preeclampsia one month earlier. There was no evidence of trauma. She was living at home with her parents and one-month-old daughter. Family history was significant for unprovoked deep vein thrombosis (DVT) and pulmonary embolism (PE) in her father at age 44, managed with apixaban. She denied taking any medications and had no smoking history. Initial observations revealed a well-nourished, well-developed young woman in moderate distress. Physical examination showed tachycardia at 160 beats per minute, mild right upper extremity swelling, and tenderness on palpation. A small mass was appreciated over the right thyroid along with right neck swelling with no overlying skin changes or cervical lymphadenopathy. Initial sonographic study revealed diffuse right internal jugular, axillary, basilic, brachial, and subclavian vein thrombosis (Figure 1).

**Figure 2:**
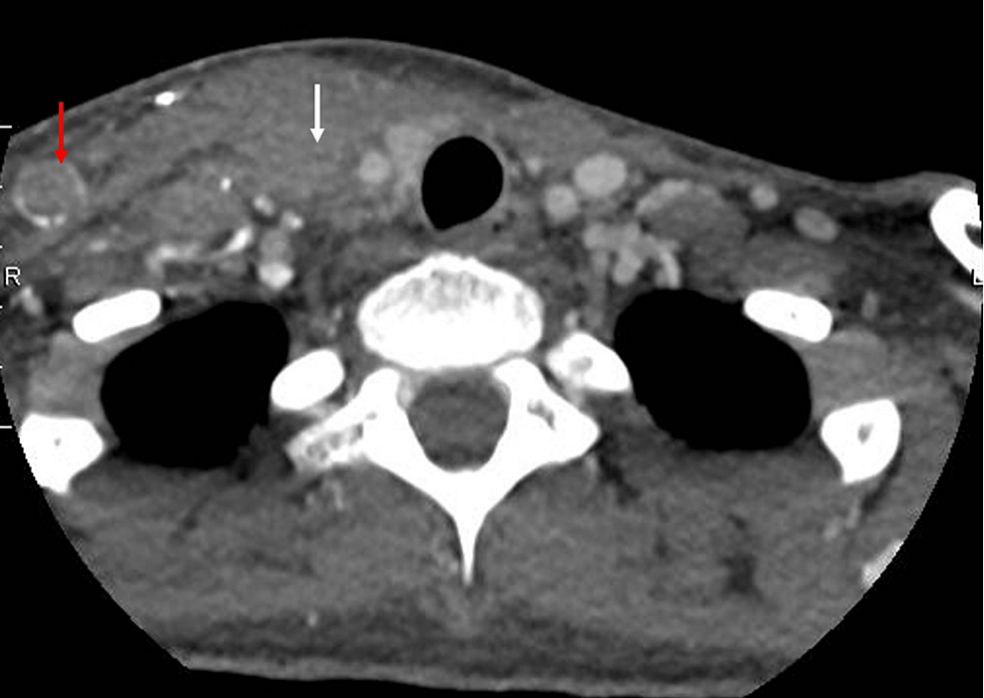
Computed tomography scan of head and neck demonstrating occlusion of the right internal (white arrow) and external (red arrow) jugular veins.

**Figure 3:**
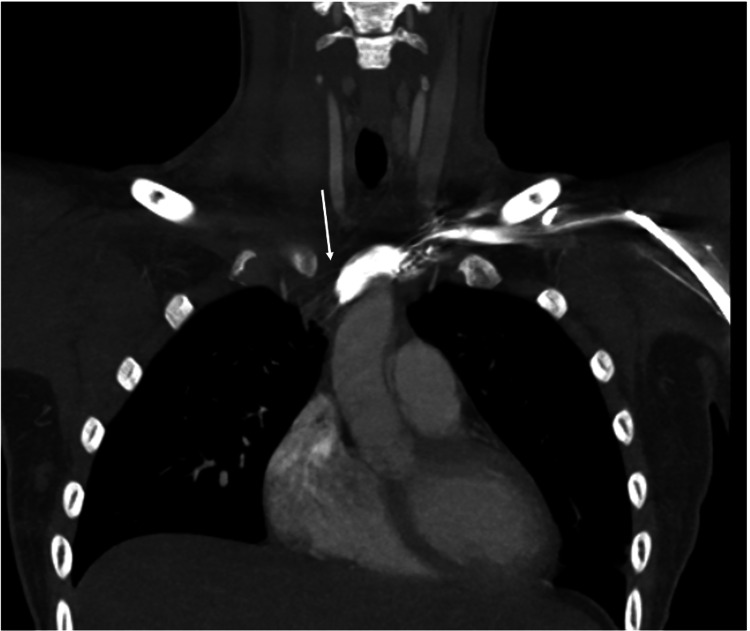
Thrombosis of innominate vein (white arrow).

Further imaging workup using computed tomography (CT) scan revealed DVT of the right brachial, basilic, cephalic, axillary, subclavian, and innominate veins without evidence of DVT in the left upper extremity (Figures 2-3).

**Figure 4:**
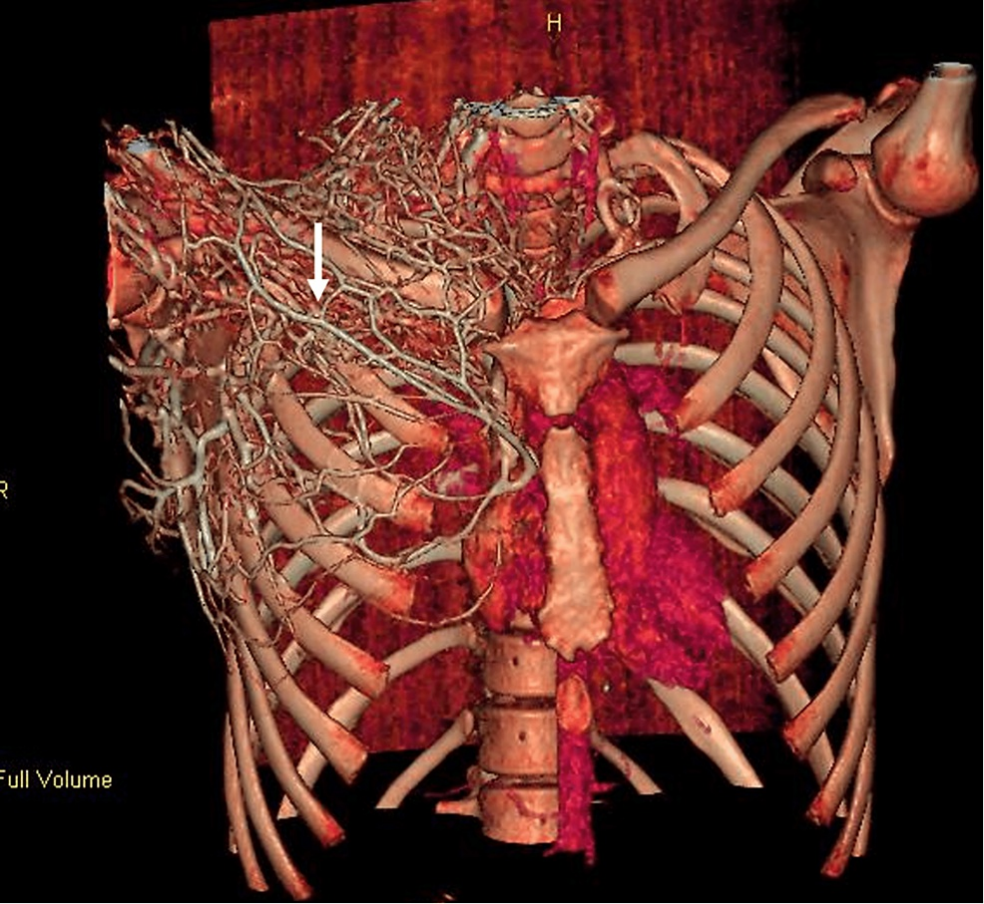
Computed tomography scan of the chest demonstrating numerous collateral vessels along the right neck and right anterior chest (white arrow).

**Figure 5:**
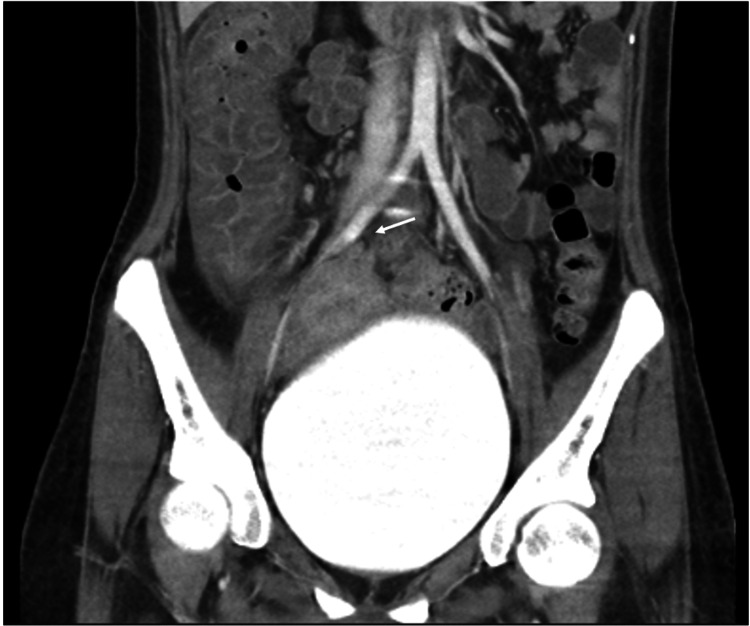
Computed tomography scan demonstrating thrombosis of the left common iliac vein (white arrow).

Prominent distention of the right internal jugular vein with surrounding fat stranding was also appreciated. Pulmonary emboli involving segmental and subsegmental branches of the bilateral lower lobes with left lower lobe pulmonary infarct were seen. Numerous collateral veins were seen along the right side of the neck and right anterior chest wall, as well as thrombosis of the left common and external iliac veins (Figures 4-5). 

Echocardiogram showed normal size and systolic function of the right ventricle; however, there was an abnormal ventricular septal motion consistent with right ventricle pressure and volume overload. Routine blood work and thyroid panel were in the normal range except for microcytic anemia with reactive thrombocytosis. A coronavirus disease 2019 (COVID-19) polymerase chain reaction (PCR) test was negative. Thrombophilia screening (paroxysmal nocturnal hemoglobinuria, antiphospholipid, anti-cardiolipin, anti-beta 2 microglobulin, and lupus anticoagulant antibodies) was normal except for a slightly decreased level (76%) of antithrombin III (ATIII), indicating a possible acquired ATIII deficiency (commonly seen after extensive thrombosis). Repeat ATIII level performed three months later was in the normal range.

Given the patient’s significant clot burden and the history of DVT/PE in her father at a young age, the need for lifelong anticoagulation despite the clot being provoked postpartum was discussed at the follow-up. Enoxaparin was used initially, followed by apixaban 0.5 mg twice daily one month after discharge. Ampicillin/sulbactam was also given for right jugular vein thrombophlebitis. Due to limited data on the safety and effectiveness of anticoagulation during pregnancy, she was counseled on using reliable contraception and to consider switching medications for future pregnancies [5]. At a follow-up 12 months later, the patient was asymptomatic, with sonogram showing no upper or lower extremity DVT. She had an elective abortion 10 months after presenting to our service and subsequently had a copper intrauterine device placed.

## Discussion

Thromboembolic events are estimated to occur in two per 1000 deliveries, with venous events (80%) being more common than arterial (20%). VTEs account for an estimated 10% of all maternal deaths; therefore, they are important to diagnose and manage. The risk of VTE is 20-fold higher in postpartum patients as compared to the normal population. Having a history of cesarean section further increases this risk [1]. The body adapts to the potential for high-volume blood loss during the peripartum period by shifting to a hypercoagulable state through upregulation of procoagulants, downregulation of anticoagulants, and decreased fibrinolysis. While these changes offer a protective advantage to the mother and fetus against bleeding, they can have deleterious consequences in the form of VTE [1].

Preeclampsia poses a five-fold increase in VTE risk than the normal pregnant population. The risk of VTE in preeclampsia may vary depending on the stage of pregnancy and the severity of preeclampsia. The prothrombotic effects of preeclampsia are more pronounced in the postpartum period. It is postulated to be due to endothelial dysfunction, increased expression of procoagulant factors, decreased anticoagulation, and increased platelet activity. In preeclampsia, impaired remodeling of the spiral arteries leads to hypoxia of trophoblast cells, oxidative stress, and a systemic inflammatory response with activation of coagulation [6]. Endothelial dysfunction may contribute to increased exposure of sub-endothelial tissue factor and impaired activated protein C anticoagulant activity. This, along with an increased expression of adhesion molecules such as ICAM-1, may promote the adhesion of inflammatory cells and increased release of endothelial extracellular vesicles (EVs). Changes in circulating platelet-derived microparticle (MP) and extracellular vesicle (EV) profiles may increase VTE risk. Placental-derived factors in preeclampsia also lead to endothelial damage [7].

Upper extremity DVTs are rare, occurring in the axillary or subclavian veins. They account for 1-4% of all DVTs [8]. They are commonly associated with thrombophilias, cancer, ovarian hyperstimulation, assisted reproductive therapy, and abnormalities of thoracic outlet anatomy. It can also be associated with strenuous activities of the upper extremity, including repetitive overhead movements, weightlifting, and rowing (termed Paget-Schroetter syndrome). UEDVTs commonly present with upper extremity pain and swelling and are diagnosed with ultrasound, similar to LEDVTs. They also can lead to PE and chronic venous insufficiency [9]. Our patient presented with typical symptoms of UEDVT, and sonogram showed extensive thrombosis of the right upper extremity veins. The combination of preeclampsia, cesarian section, and postpartum status significantly increased her risk for this complication. To our knowledge, extensive upper and lower extremity DVT with PE and this combination of risk factors has not yet been reported. Further studies are required to determine if there is an association between these two.

For the management of acute DVT in the postpartum period, unfractionated heparin or low molecular weight heparin is recommended, as was used in our patient. Warfarin may also be used with concurrent use of heparin for the initial five days until an international normalized ratio of 2-3. This should be continued for at least three to six months. Following one episode of VTE, prophylactic anticoagulation may be indicated in future pregnancies and may be accomplished using low molecular weight heparin. This is not recommended for all pregnant women, however, and limited data exist regarding its efficacy [10].

## Conclusions

Pregnancy, preeclampsia, and cesarean section increase the risk of DVT. Pregnant patients with a history of DVT should receive prophylactic anticoagulation during future pregnancies and postpartum. Furthermore, clinicians should include UEDVT in the differential diagnosis of postpartum neck pain and swelling.
